# Three-dimensional (3D) morphology of Sansha Yongle Blue Hole in the South China Sea revealed by underwater remotely operated vehicle

**DOI:** 10.1038/s41598-018-35220-x

**Published:** 2018-11-20

**Authors:** Tiegang Li, Aiping Feng, Yanxiong Liu, Zhenhong Li, Kai Guo, Wenzheng Jiang, Jun Du, Ziwen Tian, Wenxue Xu, Yang Liu, Yanru Wang

**Affiliations:** 1 The First Institute of Oceanography (FIO), State Oceanic Administration (SOA), Qingdao, 266061 China; 2 School of Engineering, Newcastle University, Newcastle upon Tyne, NE1 7RU UK; 3 Department of Policy, Law and Island’s Rights and Interests, State Oceanic Administration (SOA), Beijing, 100860 China

**Keywords:** Blue Hole, Hole Wall, Multibeam System, Steep Faces, Side Holes, Physical oceanography, Geomorphology, Geophysics

## Abstract

The Sansha Yongle Blue Hole (SYBH) is the deepest blue hole found anywhere to date. Study of the SYBH can provide insight into the interactions between hole wall morphology and many geological/hydrological mechanisms. A comprehensive investigation of the SYBH was carried out for the first time in 2017 using a professional-grade underwater remotely operated vehicle (ROV) to obtain accurate depth and three-dimensional (3D) topographic data. The SYBH resembles a ballet dancer’s shoe and has a volume of ~499609 m^3^. The observed deepest portion of the SYBH is at 301.19 m below the local 10-year mean sea level. The cave bottom laterally deviates from its entrance by 118 m at an azimuth of 219 degrees. The cave entrance is shaped like a comma and has an average width of 130 m; the widest part is 162.3 m wide, while the narrowest part is 26.2 m wide and is at 279 mbsl (meters below sea level). The 3D topography of the SYBH and underwater photography revealed two large transitions at ~76 to 78 mbsl and at 158 mbsl, indicating that the initiation of the blue hole was likely a step wise process and that the hole wall morphology was subsequently remolded through a paleo-sea level stillstand (at or near Younger Dryas). The topographic data also indicated that the blue hole is situated within an isolated environment with no water or material exchange with the outside open sea.

## Introduction

Ocean blue holes represent important natural world origin sites and geological wonders. These holes have high scientific value for researching problems related to the Earth’s geological evolution, marine paleontological evolution and climate-related issues^1–4^. The topography is the basic feature of a blue hole and is closely related to the blue hole origin, the ecosystem and water exchange^5–8^. The Sansha Yongle Blue Hole (SYBH) is the deepest known blue hole in the world^9^ and has participated in the formation and evolution of the islands and reefs in the South China Sea. This blue hole has a unique structure and geographical position and contains significant scientific information. Investigations of the SYBH are helpful for further exploring the mechanisms of blue hole genesis, examining the relationships between sea level changes and climate changes in the South China Sea, revealing unique biological community structures and ecosystem maintenance mechanisms, and providing services for marine scientific research, the environmental protection of blue holes and cultural communication.

The SYBH (N16°31.5′, E111°46.1′) located at the eastern end of the Yongle Atoll of the Xisha (Paracel) Islands, China, is ~7 km and 70 km from Jinqing Island and Yongxing Island, respectively, and ~400 km south of Sanya. Chinese fishermen have utilized the blue hole since as early as the Tang and Song Dynasties^10^. Equipped with a water level gauge (WLG) and a two-dimensional scanner on a small remotely operated vehicle (ROV) in March 2016, researchers from the Sansha Ship Course Research Institute for Coral Protection found that the cave was not a strictly vertical body. They obtained an estimated hole depth of ~300.89 m relative to the instantaneous sea surface, which is far deeper than other known blue holes around the globe^9,11,12^, including the Bahama blue hole (Dean’s Blue Hole, ~202 m), the Egyptian blue hole (Dahab Blue Hole, ~130 m), the Honduras Belize blue hole (Great Blue Hole, ~125 m) and the Malta Gozo Blue Hole (~60 m). Therefore, the SYBH is the world’s deepest ocean blue hole known to date^13^.

The preliminary survey results from the Sansha Ship Course Research Institute for Coral Protection revealed that the SYBH is not strictly a vertical cave. In the survey, the bottom of the blue hole could not be seen from the roof^11,12^, and two pronounced northward turnings and tilts were observable from 90 mbsl (meters blow sea level) to 158 mbsl, raising concerns regarding accurate topographic shape determination due to the limitations of existing underwater navigation technologies in caves. In a previous scientific expedition in 2016, the ROV could not be positioned in the lower part of the hole. The spatial structure could not be surveyed, and the ROV could not be confirmed to be at the cave bottom; therefore, accurate and reliable 3D topography data for the SYBH were not obtained.

In 2016 and 2017, Chinese researchers performed several scientific investigations in and around the SYBH to establish a safe shipping lane around the coral reef and determine the initial shape of the blue hole and its surrounding marine environment. Based on these investigations, a research team from the First Institute of Oceanography (FIO), State Oceanic Administration (SOA), China, carried out a comprehensive survey of the SYBH for the first time using an underwater ROV equipped with a Gyro Ultra-Short BaseLine (GyroUSBL) system, a Doppler Velocity Log (DVL) system, a multibeam system, and a WLG, in addition to high-definition cameras and other advanced underwater detection equipment, from May 15 to June 5, 2017. This study effectively solved the problem of underwater positioning in the blue hole with turning and tilt. Accurate depth values for the cave bottom and complete shape data for the cave walls were obtained as a result of the successful surveying and mapping of the SYBH, which can support follow-up studies.

## Results

### Topography features of the SYBH

#### External surface topography

**Figure 1:**
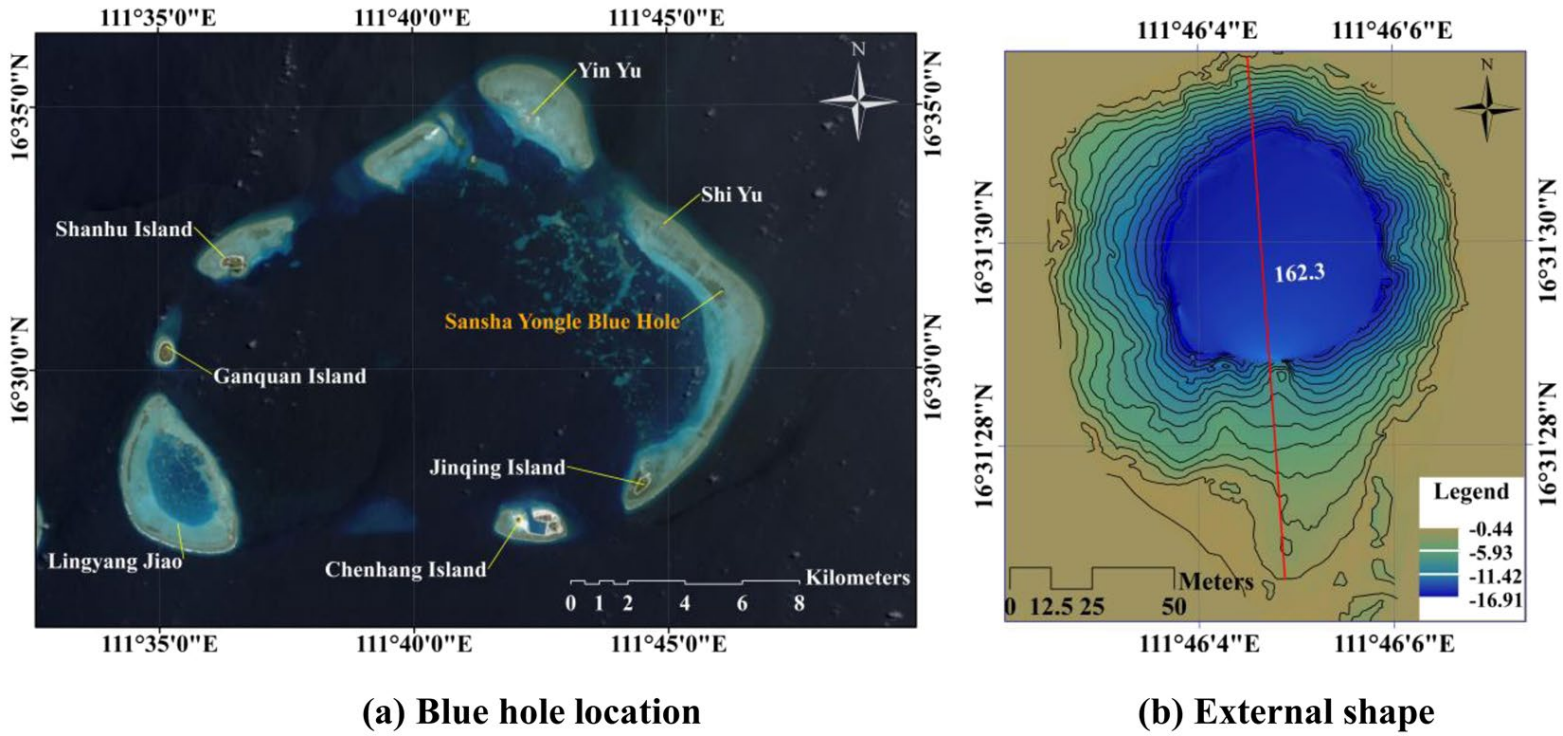
The Sansha Yongle Blue Hole (SYBH) location and external shape. The satellite image is from the NASA Landsat Program, 2016, Landsat scene LC81220492016166LGN00, L1GT, USGS, Sioux Falls, 06/14/2016.

The SYBH is located within the reef representing Jinqing Island, which is shaped like a comma. Although surrounding sediment is abundant, the ecological environment is composed of fragile coral reefs. The surrounding reefs are ~0.5 m below the local mean sea level; thus, the reefs are underwater most of the time, exposed only occasionally during the spring low tide period. The average width of the entrance of the blue hole is 130 m, and the maximum width of the entrance is ~162.3 m; the azimuth of the maximum width is ~357 degrees (Fig. 1), which is approximately north-south oriented.

A topographic transition within the blue hole entrance was observed at ~15 mbsl. The shallower section is dish shaped and gradually narrows from the surrounding sides of the entrance toward the center downward. The average slope of the edge is ~23 degrees. As shown in Fig. 1(b), the slope along the southwestern side of the entrance has a gentler slope of 15–29 degrees compared with that of the northeastern side, which has a slope of 20–33 degrees around the entrance. The slope along the edge of the blue hole gradually increases downward; at ~15 mbsl, however, the slope around the rim becomes very steep (90 degrees) and forms a vertical wall.

#### Internal surface topography

**Figure 2:**
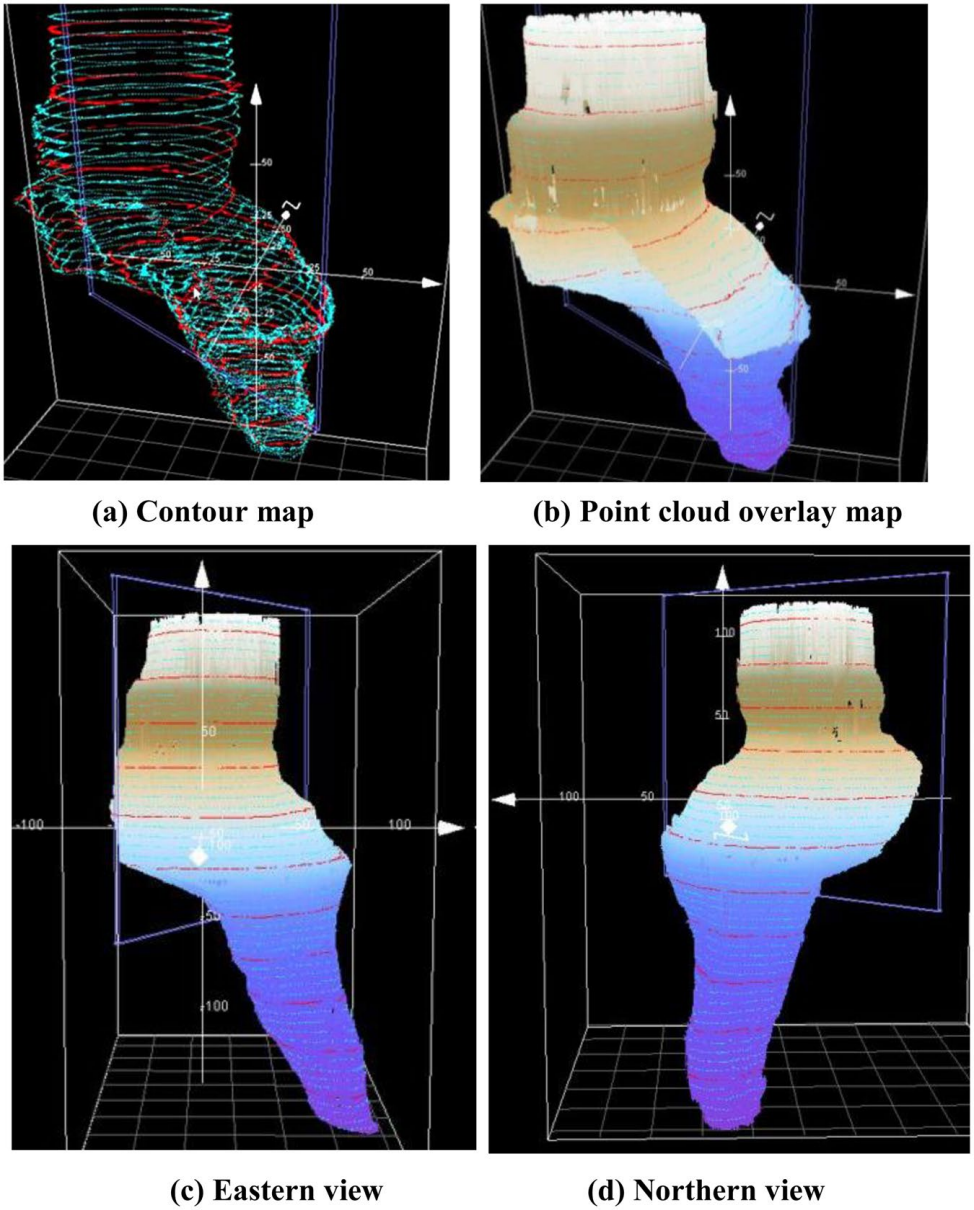
3D morphological map. The contour intervals of the green intermediate contour lines and the red index contour lines are 5 and 25 m, respectively.

An ROV platform was adopted to measure the shape of the cave inside the SYBH. By carrying acoustic positioning equipment and tilted installation of the multibeam system, scanning measurements of the blue hole from the entrance to the bottom covering all of the side holes were obtained. Subsequently, point cloud data of the entire body of the blue hole were obtained after postprocessing the multibeam scanning data. The morphological characteristics of the blue hole are shown in Fig. 2.

**Figure 3:**
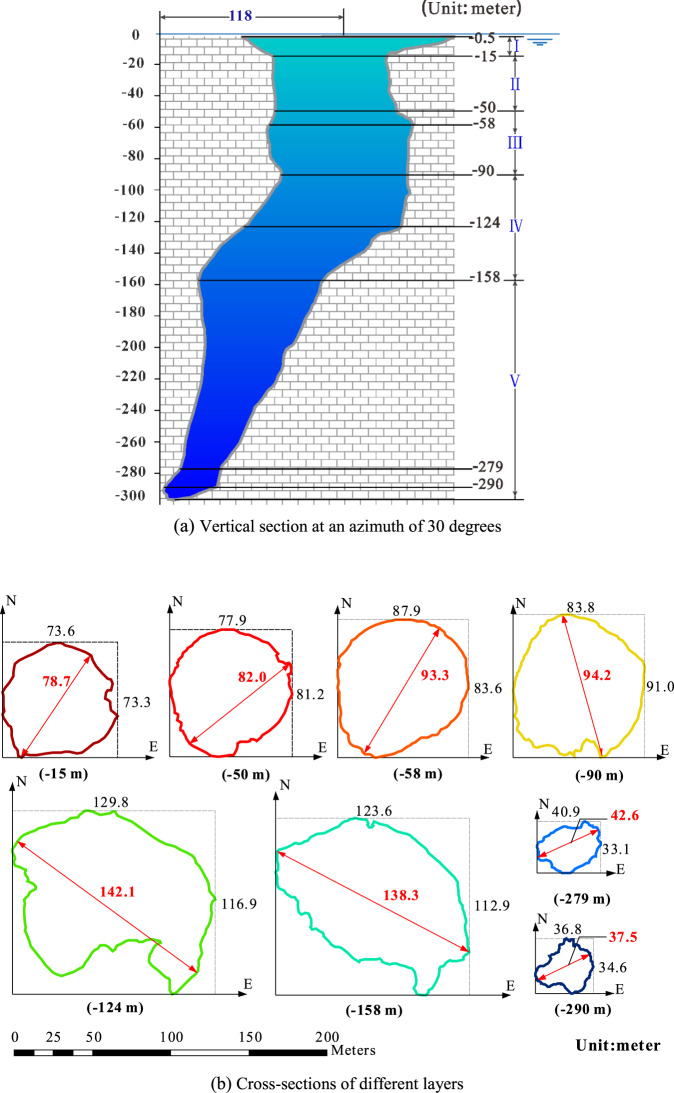
Vertical section at an azimuth of 30 degrees and cross-sections of different layers. Note that the numbers in red indicate the width at the corresponding layers.

The overall shape of the internal blue hole structure resembles a ballet dancer’s shoe. Figure 3 shows that the blue hole is not simply a vertically downward-opening cavern and that obvious, distinct sections exist. The horizontal distance between the center point of the hole entrance and the tip at the hole bottom is ~118 m (see the vertical section along the azimuth of 30 degrees in Fig. 3).

At the end of the entrance of the hole, a steep, vertical wall is evident around the center, extending to ~50 mbsl; beyond this depth, the morphological features change more rapidly. Pronounced turning occurs from 90 mbsl to 158 mbsl, and the hole extends downwards along an azimuth of 30 degrees. The cave width continually narrows to the bottom, with a minimum width of only ~26.2 m at the bottom. According to obvious vertical changes in the shape of the inner wall of the cavern, the blue hole can be divided into 5 sections, as follows (from top to bottom).

1. Cave I (entrance section) (h < 15 mbsl): The average width of the entrance is 130 m. The maximum width is ~162.3 m, and the minimum width is ~130 m. The entrance extends ~40 m from the center to the southeast. The hole entrance gradually narrows from the bottom of the reef to the center and forms a vertical wall at ~15 mbsl.
2. Cave II (15 mbsl ≤ h < 50 mbsl): The steep cave wall forms an approximately cylindrical surface, and the hole extends downwards almost vertically with an average width of 80 m.
3. Cave III (50 mbsl ≤ h < 90 mbsl): The hole extends vertically downwards. From the top to the bottom, the hole gradually widens and then becomes narrower, thereby forming a bulge in the middle of the section. The maximum hole diameter is ~93.3 m at 58 mbsl. At the bottom of this section, the average hole diameter is ~80 m.
4. Cave IV (90 mbsl ≤ h < 158 mbsl): This section spans ~70 m in depth and exhibits complex morphological changes. At 124 mbsl, the cave gradually begins to open toward the northeast following a vertically opening trend, and the south wall shows a pronounced uplift with a decrease in slope, forming a gentle “shoulder”. The central cavity is obviously enlarged and is characterized by a decrease in width from the southwest to the northeast. Meanwhile, the vertical width increases to a maximum of 142.1 m.
5. Cave V (−158 m ≤ h): The cave continues to open along a northeast direction with a depth range of ~140 m. The hole width gradually decreases and forms a funnel shape. At 279 mbsl, the cavern narrows to a minimum width of only 26.2 m. After this point, the cave widens slightly; at 290 mbsl, the hole is 37.5 m wide. Below 290 mbsl (the bottom of the cavern), the hole exhibits a tilted “bowl” shape.

#### Hole wall features

1. **Side hole:** The internal walls of the SYBH are mainly composed of reef limestone, and their morphology is complex. Multiple vertical side holes are apparent along the hole wall. The two largest side holes were found at ~33 mbsl and 43 mbsl; their images and geomorphology are illustrated in Fig. 4A,B, respectively. The shallower side hole exhibits an irregular shape. The radius of this side hole is ~2 m, and the hole wall is gently concave (i.e., like a hemisphere). The surface of the side hole is smooth, and the estimated maximum inward depth is ~3 m. The deeper side hole is larger and, similar to the shallower side hole, exhibits an irregular shape. Its horizontal length is ~6 m, and its vertical height is ~5 m; its estimated inward depth is ~2 m. The shape of the side hole is complex with a rough wall surface. The formation mechanisms of the side holes require further study with regard to other physicochemical characteristics.

**Figure 4:**
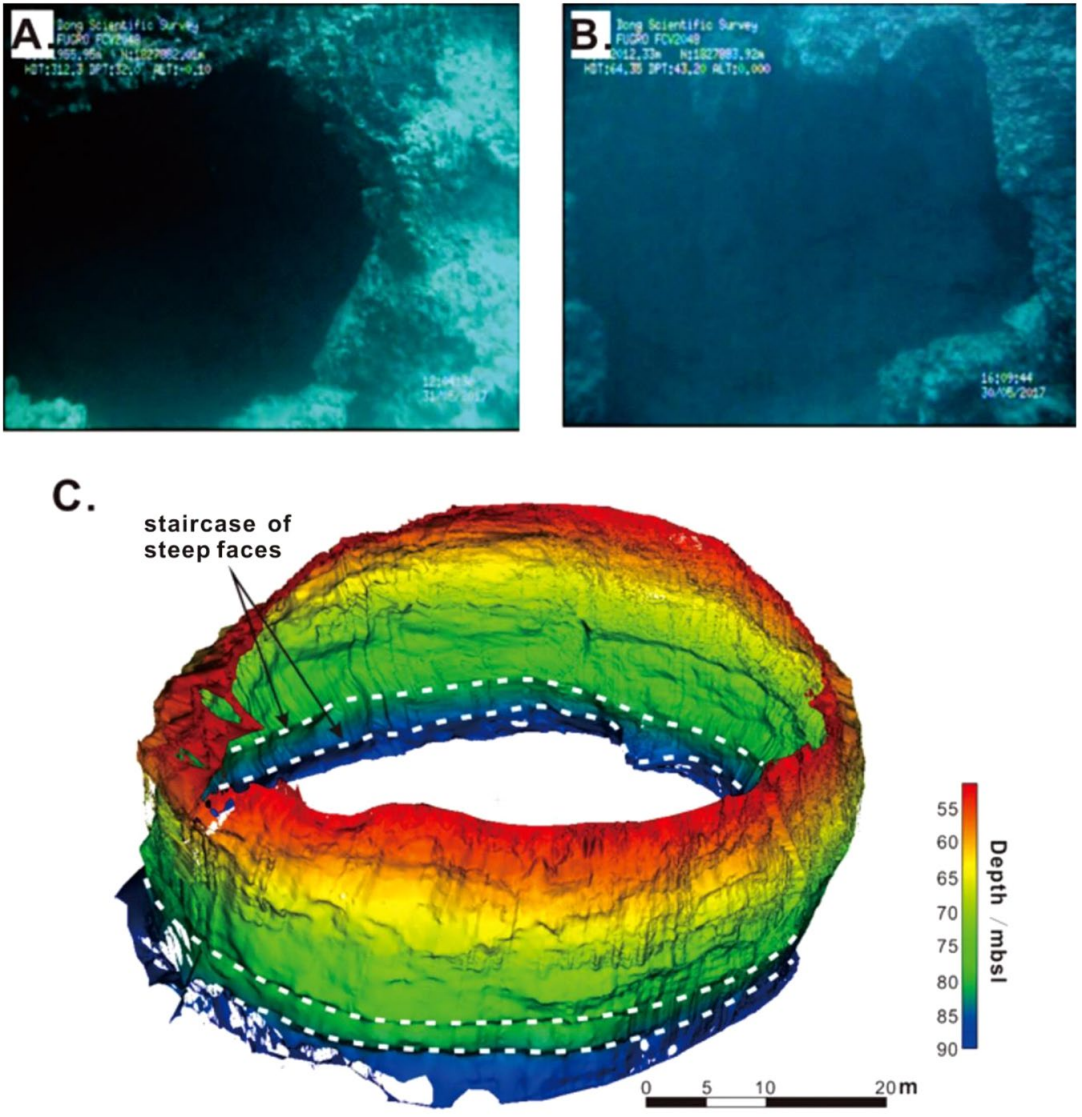
Underwater photographs of side holes at (**A**) 33 mbsl and (**B**) 43 mbsl, and (**C**) hole wall 3D topography illustrating staircase of steep faces at ~76 mbsl.
2. **Staircase of steep faces:** At the transition from Cave III to Cave IV, we observed an ~2-m-wide zone composed of a staircase of steep faces (Fig. 4C). This feature occurs at ~76 to 78 mbsl and comprises 2 stairs that extend up to 1.5 m from the surrounding hole wall. The existence of the side holes and the staircase feature along the hole wall emphasize the complexity of the internal morphology of the blue hole.

### Depth of the SYBH

#### Chart datum

The water depth is usually represented as the vertical distance relative to the theoretical depth datum. However, the local mean sea level is commonly used as the depth datum in areas far from the mainland. The local 10-year mean sea level is used as the depth datum for the SYBH, which was determined via *in situ* tide level observations together with long-term tide gauge observations.

The SYBH is located in the open South China Sea, and the mean sea level is nearly the same in this area^14^. In this open sea, a long-term tide gauge station named Xisha is located 60 km northeast of the blue hole. The 10-year mean sea level was calculated based on the Xisha tide observations. To transfer the 10-year mean sea level to the blue hole, an RBRduo WLG was placed on Yinyu Island 7 km north of the blue hole for continuous observation from February 25 to May 31, 2017, and another RBRduo WLG was simultaneously placed at the entrance of the blue hole for continuous observation from May 19 to May 30, 2017. The 3-month mean sea level on Yinyu Island is only 2 cm higher than the 10-year mean sea level at the Xisha tide gauge station; therefore, the sea level is nearly the same.

According to the WLG data analysis, the elevation of gauge zero on Yinyu Island is 0.906 m lower than the 10-year mean sea level and 0.396 m lower than the elevation of gauge zero on the blue hole. Therefore, the elevation of gauge zero on the blue hole is 0.510 m lower than the 10-year mean sea level and its calculated accuracy is better than 0.01 m.

#### Depth results

**Figure 5:**
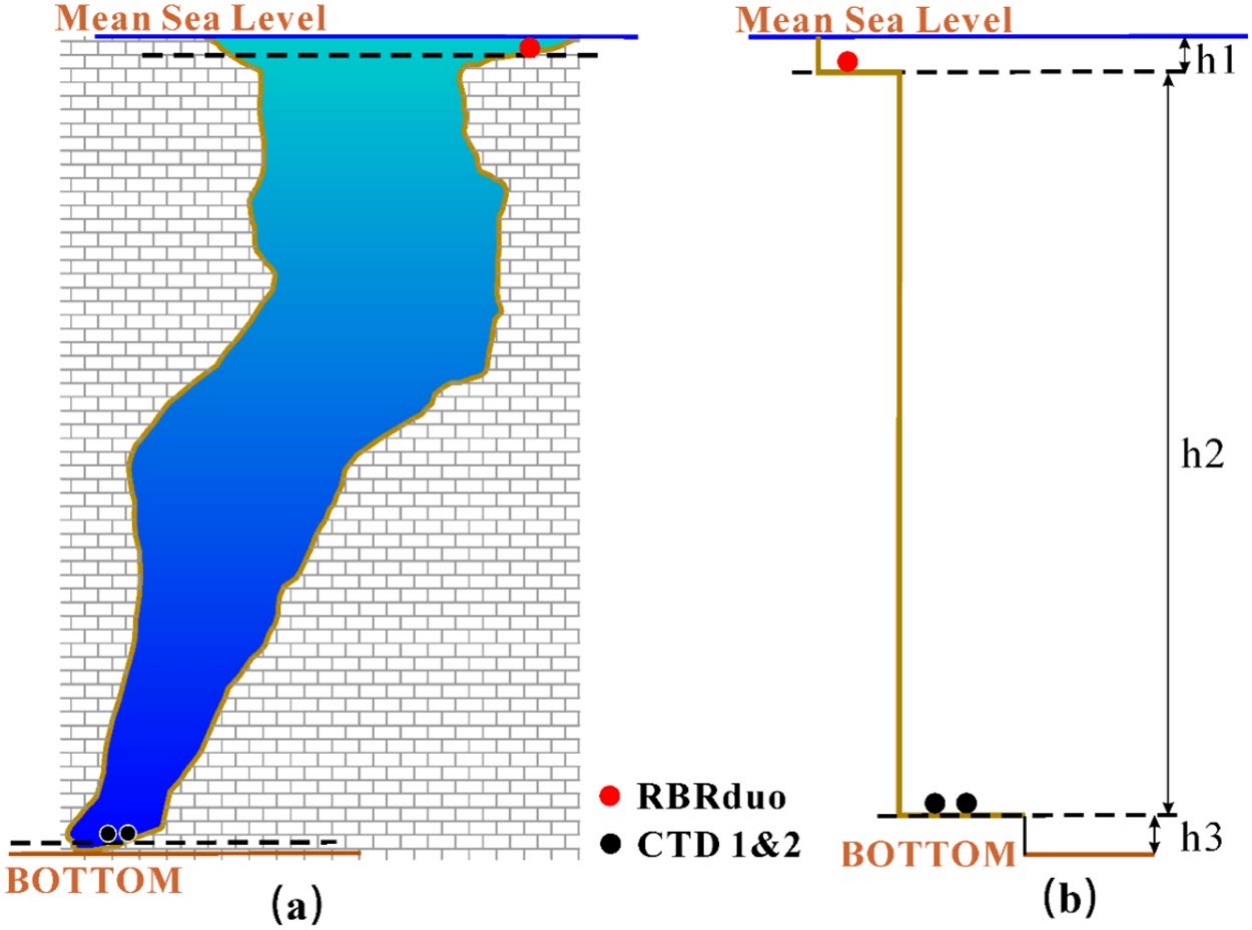
Blue hole depth and the corresponding surveying method.

Four WLGs, one GyroUSBL system, one DVL system and one multibeam system were employed to measure the depth of the blue hole. In this study, the depth of the SYBH was used to represent the depth of the deepest point within the blue hole. One WLG was placed near the entrance of the blue hole (RBRduo in Fig. 5), one WLG was loaded on the ROV, and the other two were placed at the bottom of the blue hole (CTDs 1 and 2 in Fig. 5). The observation period spanned from May 23 to May 31, 2017. The sampling interval of the anchored WLG was 10 minutes, while the sampling interval of the WLG mounted on the ROV was 1 second. The WLGs on the ROV and GyroUSBL provided real-time instantaneous positions of the ROV for multibeam system surveying. The bathymetric results are mainly based on the WLG data, and the multibeam measurement results with GNSS results were used for auxiliary analysis.

Determination of the depth of the blue hole involved determining the depths of three components, as shown in Fig. 5. The first part, h1, is the height difference between the location of the RBRduo and the 10-year mean sea level and was found to be 0.51 ± 0.01 m using tide gauges and GNSS positioning. The second part, h2, is the depth difference between the RBRduo and CTD 1, which was determined to be 299.92 ± 0.0067 m by integrating the pressure difference and seawater density. As a check, the system bias is only 0.016 m between CTD 1 and CTD 2. The third part, h3, is the height difference between CTD 1 and the deepest bottom depth. The fitted value is 0.76 m based on point cloud data from the multibeam system. The fitted accuracy is ~0.02 m. Therefore, the final depth of the blue hole is 301.19 ± 0.023 mbsl (in reference to the 10-year mean sea level).

## Discussion

### Step wise initiation of the blue hole

The Caves I to III are nearly vertical, and the next cave below (Cave IV) is characterized by the greatest cave section area among the caves (Fig. 3). These findings suggest that these caves were likely shaped by the collapse of the roof; analogs have been widely observed in many coastal sinkholes and reefal blue holes^15^. This hypothesis is supported by the distribution of the collapsed sediments (white muds) observed through the underwater photography: these sediments were predominately distributed within Caves IV and V, especially at the platform of Cave IV, but were seldom observed within Caves I to III. The bottom cave (Cave V) has a smaller cross-sectional area than the caves above, and its orientation differs from those of Caves I to IV (Fig. 3), indicating that this cave likely originated and developed via a mechanism and over a time span different from those of the other caves. The abovementioned hypotheses are based solely on the 3D topography of the blue hole; since the genesis of a blue hole is a complicated process associated with several geological, hydrologic and biological regimes, more evidence is needed to test these hypotheses.

### Evidence of a previous stillstand

The staircase of steep faces appears at the transition zone from the steep vertical hole (Caves I to III) to the larger middle hole (Cave IV). As reported by previous researchers^16^, such geometry is indicative of an active environment, which is presumably associated with the stay of sea level in the location of the SYBH in our study (i.e., stillstand). This hypothesis is supported by the postglacial sea-level curves that have been reported within coastal waters of China^17–22^, which indicate a considerable slowing down of sea level rise during the Younger Dryas (YD) event and potentially a long-term sea level stillstand at ~70 mbsl. The depth of YD stillstand sea level is consistent with the depth of the observed staircase features (~76 to 78 mbsl), given that regional subsidence can likely explain the discrepancy between the two depths. The hypothesis is also supported by the existence and locations of the side holes. Since the development of side holes requires a subaerial environment in the vadose zone^15^; the fact that all the side holes in the Xisha Yongle Blue Hole were observed at depths shallower than ~60 mbsl suggests the long-term exposure of Caves I to III. Thus, the sea level position must remain lower than the side holes for a long time.

### The enclosed (isolated) environment

The investigation ascertained the depth and morphology of the SYBH, i.e., 301.19 m in depth and no horizontal phreatic conduits, which are the main causes of the stagnant water condition deep in the cave. Tidal currents and surface waves can only reach a maximum depth of ~60 mbsl in an enclosed cave^23,24^; thus, the deeper part of the SYBH is beyond this depth. On the other hand, no direct conduit was found on the wall connecting the cave with the open sea, and the bottom of the cave was silted with sediment. All these features contribute to the minimal exchange of deep water in the cave with outside water, forming an isolated environment. The deep water, below 80 m, in the cave shows no sign of directional movement and is anaerobic, which is consistent with our morphology observations.

## Methods

**Figure 6:**
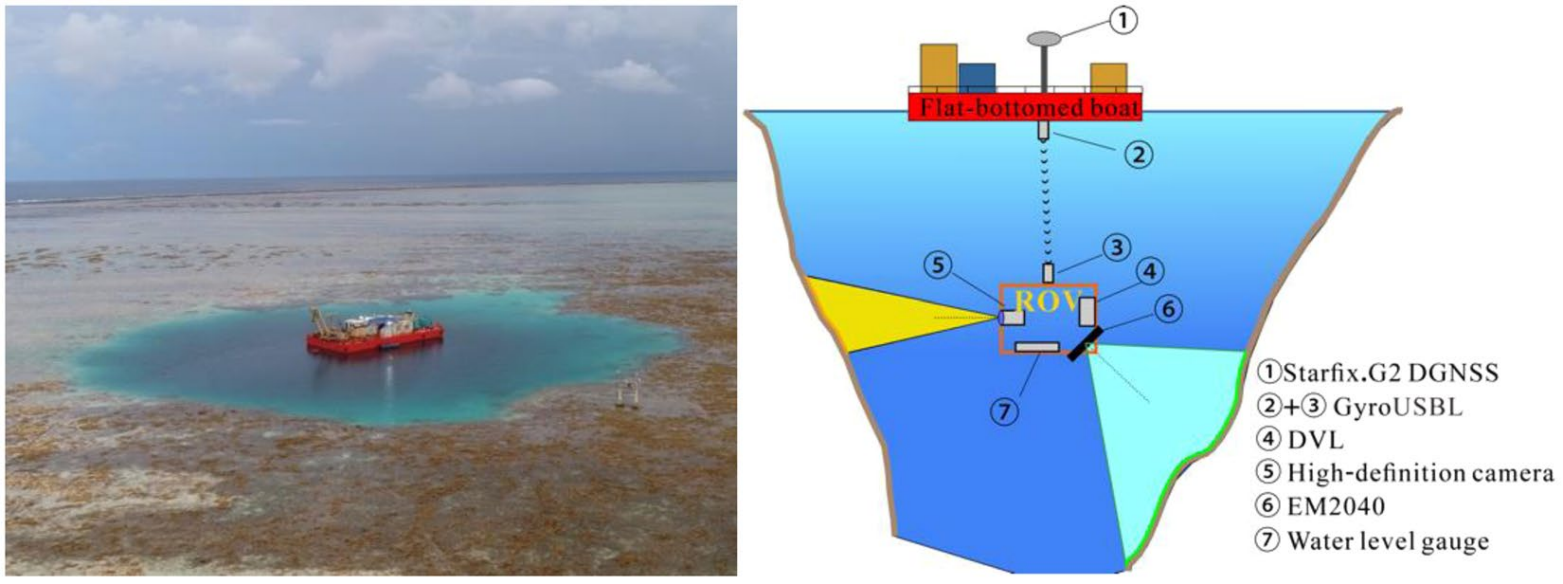
The device carrier and the surveying techniques.

The blue hole entrance is located within a coral reef. The water depth of the reef is only 0.5–0.8 m on average, and the spring high tide lasts ~2 hours with a water depth of only ~1 m. At the spring low tide, the hole entrance is exposed, and the water depth is not sufficient to permit large ships access to the hole entrance. Due to the location particularities and the shape complexity of the blue hole, two methods were creatively employed in the investigation, as shown in Fig. 6. The first method used a flat-bottomed boat constructed as the device carrier. The second method is the underwater integrated navigation system designed to position the ROV in real-time.

### The device carrier

To transport large survey equipment into the blue hole without damaging the coral reef, the expedition team from the FIO designed and constructed a flat-bottomed boat (25 × 18 m). At high tide, the team towed the boat to the center of the blue hole entrance and anchored it to a selected point on the surrounding reef. The boat was then used as the basic carrier for both personnel and equipment during the investigation process to provide a stable platform for launching underwater instrumentation and to minimize impacts on the surrounding environment (Fig. 6).

The GNSS and transponder are mounted on the device carrier for positioning. The underwater survey used an FCV2000D professional-grade ROV with an external size of 3.0 × 1.7 × 1.7 m and a maximum operating depth of approximately 3000 m^25^. It was equipped with a DVL system and a GyroUSBL system in addition to a high-definition camera, a multibeam sounding system, and a WLG, as shown in Fig. 6. The GyroUSBL, DVL equipment and WLG were mainly used for underwater positioning and navigation^26^. The EM2040 multibeam sounding system was used to measure the shape of the cavern^27,28^, and the underwater high-definition camera was employed to capture high-definition video of the hole wall.

### Positioning and navigation

The positioning system utilized on board the operational platform was a Fugro Starfix.G2 differential GNSS (DGNSS) system. This system was mainly employed to determine the position of the ship and then the transponder’s position. After verification, the horizontal accuracy of the system was 0.1 m, and the vertical accuracy can reach 0.2 m. The ROV underwater positioning system uses the “GyroUSBL + DVL + WLG” model in which both high-precision inertial navigation equipment and depth sensors are used for location assistance and real-time corrections based on USBL underwater positioning. During ROV motion, the ranging accuracy of the model is 0.015 m, and the positioning accuracy is 0.1% of the movement distance. The system provides positioning and attitude information at up to 5 Hz. Compared with general USBL underwater positioning, this model can provide higher-accuracy real-time movement information of an ROV^26,29,30^.

In addition, *in situ* navigation and data recording were conducted using the Starfix Fugro Suite integrated navigation system. This system can combine various navigational equipment data, determine the position and attitude of an ROV platform and display real-time target positions and relative underwater relationships, thereby providing position, attitude, heading and depth information for a multibeam system and other investigation equipment^31^.

### Tidal measurements

To obtain real-time local tide data in addition to collecting other relevant tidal information, our investigation from February to June 2017 established several temporary tide stations to conduct real-time monitoring of the local tidal levels in the waters surrounding the blue hole. Therefore, the tidal level data analysis in this study is mainly derived from the *in situ* measured data obtained at the temporary tide gauge stations. The coordinates of the automatic tide gauge zero point were obtained using a GNSS technique. The GNSS data-processing results show that the height accuracy of the tide gauge zero point is better than 3 cm.

The local mean sea level in the blue hole area was calculated according to the tidal measurements and used as the chart datum for the depth measurements^32^. Sufficient tide measurements can provide reliable references for the design of ROVs and multibeam systems, data post processing and tidal corrections.

### Three-dimensional topography surveying

The three-dimensional topography survey of the blue hole consisted of two parts, i.e., above 15 mbsl using the unmanned SeaRobot ship equipped with a multibeam system^33^ and below 15 mbsl using the professional-grade ROV equipped with a multibeam system. To achieve full 3D coverage of the hole body, the multibeam sounding system was installed on the ROV with a tilt of 30 degrees to scan for different layers. Sweeping scans were carried out at 15 mbsl, 50 mbsl, 90 mbsl, 110 mbsl 200 mbsl and 280 mbsl. The sweeping scans at 50 mbsl and 90 mbsl were carried out twice. The scanning measurement data acquired below depths of 15 m were then spliced and combined with the multibeam data on board the unmanned ship acquired above 15 mbsl. Finally, the entire 3D shape of the blue hole was determined.

The primary difficulty of this approach is related to postprocessing of the measurement data because the graphical data-processing algorithm included within mainstream data-processing software mainly addresses vertical scans to the water bottom, while the internal scanning data for the blue hole involve vertically stacked layers. In addition, the blue hole is an enclosed space affected by the cavern structure and the water environment during the scanning process; furthermore, the acoustic environment, which is characterized by severe reverberation, is complex. Computerized automatic processing is subject to many mistakes and miscalculations, and meeting the surveying requirements for investigation of the blue hole is difficult. To solve this problem, we used the Starfix multibeam processing software developed by the Fugro company for data below 15 mbsl. The software provides a multibeam data acquisition and detection procedure in strict accordance with the multibeam data-processing requirements. We used the VBAProc module for tide and velocity corrections, while attitude corrections and noise filtering were performed in the SwathEdit module. Finally, the results were exported through the Workbench and displayed using a relevant digital graphical model. For parts of the region characterized by complex scanning data, manual intervention was necessary to ensure the reliability and validity of the results. Although manual verification is performed at the expense of efficiency to a certain extent, it can ensure control of the data quality and provide valid results.
