# Author Correction: Three-dimensional (3D) morphology of Sansha Yongle Blue Hole in the South China Sea revealed by underwater remotely operated vehicle

**DOI:** 10.1038/s41598-023-33980-9

**Published:** 2023-05-08

**Authors:** Tiegang Li, Aiping Feng, Yanxiong Liu, Zhenhong Li, Kai Guo, Wenzheng Jiang, Jun Du, Ziwen Tian, Wenxue Xu, Yang Liu, Yanru Wang

**Affiliations:** 1 The First Institute of Oceanography (FIO), State Oceanic Administration (SOA), Qingdao, 266061 China; 2 School of Engineering, Newcastle University, Newcastle upon Tyne, NE1 7RU UK; 3 Department of Policy, Law and Island’s Rights and Interests, State Oceanic Administration (SOA), Beijing, 100860 China

Correction to: *Scientific Reports* 10.1038/s41598-018-35220-x, published online 20 November 2018

This Article contains an error in the Abstract, where the volume of the Sansha Yongle Blue Hole is incorrect.

“The SYBH resembles a ballet dancer’s shoe and has a volume of ~499609 m^3^.”

should read:

“The SYBH resembles a ballet dancer’s shoe and has a volume of 1417149 m^3^.”

